# Effects of *Lactobacillus gasseri* CP2305 on Mild Menopausal Symptoms in Middle-Aged Women

**DOI:** 10.3390/nu14091695

**Published:** 2022-04-19

**Authors:** Daisuke Sawada, Tomonori Sugawara, Tatsuhiko Hirota, Yasunori Nakamura

**Affiliations:** Core Technology Laboratories, Asahi Quality & Innovations, Ltd., Midori, Moriya-shi 302-0106, Ibaraki, Japan; tomonori.sugawara@asahi-qi.co.jp (T.S.); tatsuhiko.hirota@asahi-qi.co.jp (T.H.); yasunori.nakamura@asahi-qi.co.jp (Y.N.)

**Keywords:** paraprobiotics/probiotics, women’s health, menopause, psychological effect, gut–brain axis

## Abstract

*Lactobacillus gasseri* CP2305 (CP2305) is a paraprobiotic that exhibits beneficial effects on the intestinal function and microbiota, and increases resistance to psychological stress. The stress response mechanism mainly involves the hypothalamic–pituitary–adrenal axis, which is influenced by the gut–brain axis. Furthermore, the gut–brain axis also communicates bidirectionally with the intestinal microbiota. Additionally, the hypothalamic–pituitary–adrenal and hypothalamic–pituitary–gonadal axes share a common route that affects both mental and health aspects in women. This double-blind, placebo-controlled, parallel-group clinical trial aimed to analyze the influence of the intake of CP2305 on mild symptoms associated with menopause. Eighty women aged 40–60 years ingested CP2305 or placebo tablets for six consecutive menstrual cycles. Assessment was based on the observation of climacteric symptoms with two validated questionnaires—the Simplified Menopausal Index (SMI) and the Greene Climacteric Scale (GCS). The results showed that CP2305 provided significant relief in the SMI total score, SMI vasomotor score, SMI psychological score, GCS total score, GCS somatic score, and GCS vasomotor score compared to the placebo. The percentage of women with symptom relief for the SMI total score was 75.0%, with 30 of 40 women in the CP2305 group, and 55.0%, with 22 of 40 women in the placebo group (*p* = 0.0594). These findings provide new insights into the function of paraprobiotic CP2305 in relieving mild climacteric symptoms in women.

## 1. Introduction

The gastrointestinal tract represents the largest interface and interaction between the host and microorganisms such as bacteria, archaea, and eukarya. This collection of microorganisms is called the enteric or gut microbiota (GM), and co-evolved with their hosts in an intricate, mutually beneficial relationship [1]. One of the most important interactions is the brain–gut interaction, which maintains both intestinal homeostasis and brain function. This interaction and its relationship with the GM have been expanded to a concept known as the “microbiota–gut–brain axis” [2,3,4].

GM is regulated via immune, endocrine, or neural pathways, and by different hypothalamic–pituitary axes, such as the hypothalamic–pituitary–adrenal (HPA) axis and hypothalamic–pituitary–gonadal (HPG) axis [5,6,7]. Both the HPA and HPG axes interact to regulate mental stress response [8]. An example is the effect of fluctuations in the gonadal hormone estradiol (E_2_) during the menstrual cycle and its effect on the HPA activity. Crosstalk between the HPG and HPA axes can lead to abnormal stress responses and neuropsychiatric disorders [8].

When menstrual periods permanently cease, women reach menopause. During this period, which can last for several years and is known as perimenopause, women experience symptoms related to fluctuations in hormonal levels. As the ovaries reduce in size, the menstrual cycle becomes irregular, and urogenital symptoms become predominant [9]. Additionally, other symptoms related to the cardiovascular system and psychological dysfunction have been elucidated [10].

In 2001, the International Menopause Society (IMS), in conjunction with other groups, proposed the Stages of Reproductive Aging Workshop (STRAW). The criteria define each stage of reproductive ageing through menopause [11]. STRAW+10 provides the basis for changes in the functioning of the HPG axis to facilitate the comparability of studies on middle-aged women and clinical decision-making [11]. The 10-year period around menopause, five years prior to and five years following menopause, is known as the “climacteric” stage. The mean age at which women reach menopause is reported to be either 50.54 or 52.1 years [12,13]. The transitional stage before menopause is referred to as “menopausal transition”. During this stage, the main complaints are symptoms of imbalance in the autonomic nervous activity (vasomotor-related symptoms) and psychological symptoms. Decreased E_2_ levels, psychosocial factors, and the age of women are reported to trigger such symptoms. The causes of these symptoms are not fully understood, but recent research suggests that the enteric flora is involved in environmental E_2_ metabolism through its role in maintaining intestinal homeostasis [14,15].

In recent years, studies regarding the implications of GM on our health have increased, and evidence suggests that they influence the host through various metabolites. Examples include prebiotics, probiotics, and paraprobiotics [16]. Prebiotics are nutrients that are degraded by the GM [17]. In contrast, according to the Food and Agriculture Organization/World Health Organization (FAO/WHO), probiotics are “live microorganisms that when administered in adequate amounts confer health benefits to the host” [18]. The terms postbiotics and paraprobiotics have been used indistinctly; however, paraprobiotics are inactivated microbial cells or cell fractions, such as peptidoglycans, teichoic acids, and surface proteins, that confer health benefits to the consumer [19], whereas postbiotics are metabolic products secreted by probiotics, such as enzymes, proteins, short-chain fatty acids, vitamins, and biosurfactants [20]. Most recently, the International Scientific Association of Probiotics and Prebiotics (ISAPP) defined postbiotics as the preparation of inanimate microorganisms and/or their components that confer health benefits to the host, including paraprobiotics [21].

*Lactobacillus gasseri* CP2305 (CP2305), a paraprobiotic originally isolated from the stool sample of a healthy volunteer, exhibits a stress-relieving activity [22,23]. The daily intake of CP2305 for 12 weeks reduced stress-associated mental and physical symptoms related to preparation for a national certification examination, attenuated stress-induced changes in the salivary cortisol concentrations, and an elevated expression of stress-responsive microRNAs in the peripheral blood [24]. These observations suggest that the administration of CP2305 may affect central nervous system functions and modulate the HPA axis; therefore, CP2305 is a *Lactobacillus* that increases resistance to psychological stress. Furthermore, it has been reported that the daily intake of CP2305 improved the clinical symptoms of patients with irritable bowel syndrome [25], altered the gastrointestinal microbiota composition, reduced pre-menstrual discomfort, and improved the quality of sleep by regulating autonomic activity [23].

Based on these prior studies, this study aimed to analyze the influence of CP2305 intake on the HPG axis and its effects on mild symptoms associated with menopause.

## 2. Materials and Methods

### 2.1. Study Setting

The present clinical trial was approved by the Institutional Review Board of the Medical Corporation Hokubukai Utsukushigaoka Hospital (protocol number 15000060) and was conducted according to the ethical standards established in the Declaration of Helsinki. All participants provided written informed consent before enrolling in the study. This study was registered with the University Hospital Medical Information Network (UMIN) Clinical Trials Registry as “a study to evaluate menopausal symptoms in middle-aged women” (UMIN000039086).

### 2.2. Study Population

This study was designed as a double-blind, placebo-controlled, parallel-group clinical trial (Table 1). The sample size calculation was performed with G*Power 3.1.9.7 (Heinrich-Heine-Universität Düsseldorf, Düsseldorf, Germany) using the F-test (analysis of variance (ANOVA) with repeated measures, within-between interaction). The effect size f was derived as 0.1429 using the small effect size (partial eta squared for repeated measures ANOVA), and the required sample size of 70 was calculated assuming an alpha error probability of 0.05 and power (1-beta error probability) of 0.80. Given the long intervention period, the dropout rate was estimated to be approximately 30%. After taking into account the 30% dropout rate, the required sample size for this study was set at 100 subjects.

The selection criteria for participants were as follows: (i) Japanese premenopausal women aged 40–60 years. The reason for the broad age range of 40 to 60 years is that menopause is usually reported to occur between the ages of 40 and 58 [26], and symptoms are reported to have individual differences [27]. (ii) Those who had no climacteric disorder and had several mild climacteric symptoms evaluated by the Simplified Menopausal Index (SMI) as 26 to 50 points. (iii) Those who had normal E_2_ production. (iv) Those who had a normal menstrual cycle (with 3–7 days of menstrual period and 25–38 days between cycles). Those with (v) a body mass index (BMI) between 18.6 and 30 kg/m^2^. Those (vi) had a fatigue (a profile of Mood Stats 2 (POMS2) fatigue score ≥ 9 points) and vigor score <12 points. Those who (vii) provided signed informed consent.

Subjects excluded when they (i) were patients with, or patients with a history of, serious disease of the liver, kidney, heart, thyroid, and internal organs or diabetes. Those with (ii) a history of ovariectomy or hysterectomy. Those who were consuming medications for menopausal symptoms. Those who (iii) had internal medical chronic diseases. Those (iv) who were constantly using medicines and functional health foods that promoted menopausal symptoms. Those who (v) were shift workers, including late-night shifts. Those who (vi) were pregnant, breastfeeding, or intended to become pregnant during the test. Those (vii) had excessive smoking or drinking habits. Individuals (viii) were judged inappropriate for the trial by the principal investigator.

The enrolled subjects were randomly allocated to either the CP2305 or placebo groups. The participants were instructed to ingest two tablets (placebo or 1 × 10^10^ CP2305 bacterial cells) once daily for six menstrual cycles. The rationale for the dosage was based on previous reports [23,28]. To assess compliance, the participants self-reported their tablet intake. During the trial, participants were asked not to consume fermented milk, foods containing live lactic acid bacteria, or other probiotic or prebiotic products.

The primary outcome was a change in the scale of the questionnaire for the assessment of menopausal symptoms over time in the CP2305 group compared with that in the placebo group. The secondary outcome was a change in the concentration of reproductive hormones during the follicular phase before and after administration in the CP2305 group compared with that in the placebo group.

### 2.3. Preparation of Tablets Containing CP2305

Both the CP2305-containing and placebo tablets were prepared using the same procedures and formulas, except for the presence and absence of heat-inactivated, washed, and dried CP2305 (1 × 10^10^ bacterial cells per two tablets). The active tablet comprised maltose, dextrin, starch, heat-inactivated lactic acid bacteria powder, and vegetable oils. The placebo tablet was similarly composed, except that the lactic acid bacteria powder was replaced with dextrin. The formula was allergen-free.

### 2.4. Questionnaires to Assess Menopausal Symptoms

Menopausal symptoms were evaluated using the SMI [29,30] and Greene Climacteric Scale (GCS) [31]. The SMI comprised 10 questions assessing vasomotor (four items: hot flashes, sweats, chills, and shortness of breath or palpitation), psychological (four items: sleep disturbances; easy excitability or irritability; worry about self-depression; and headache, vertigo, or nausea), and somatic (two items: easy fatigability, and shoulder stiffness, lumbago, or joint pain) symptoms. The SMI is frequently used to assess menopausal symptoms in women in Japan. The GCS comprises 21 questions assessing psychological, somatic, vasomotor, and sexual symptoms. The participants reported their symptoms using the SMI and GCS every two menstrual cycles during the experimental period.

### 2.5. Measurements of Serum Estradiol, Progesterone, Follicle-Stimulating Hormone, and Luteinizing Hormone

Blood was collected 3–6 days after ovulation every two cycles after the intervention. The concentrations of serum E_2_, progesterone (P_4_), follicle-stimulating hormone (FSH), and luteinizing hormone (LH) were measured by SRL, Inc. (Tokyo, Japan).

### 2.6. Measurements of Urinary Equol

Urine was collected using sampling devices before the intervention. The concentrations of urinary Equol (Equol ELISA kit; Healthcare Systems Co., Ltd., Nagoya, Japan) were measured according to the manufacturer’s instructions. Urine samples were stored at −80 °C until analysis.

### 2.7. Statistical Analysis

Statistical analysis was performed using JMP version 13.0 (SAS Japan, Tokyo, Japan). Data are presented as mean ± standard error of the mean (SEM). The changes in the questionnaire scores and the concentration of serum hormones were averaged for each menstrual cycle, and the changes were analyzed using two-way ANOVA with repeated measures. A *p* < 0.05 was considered to be statistically significant.

## 3. Results

### 3.1. Characteristics of the Subjects

**Figure 1:**
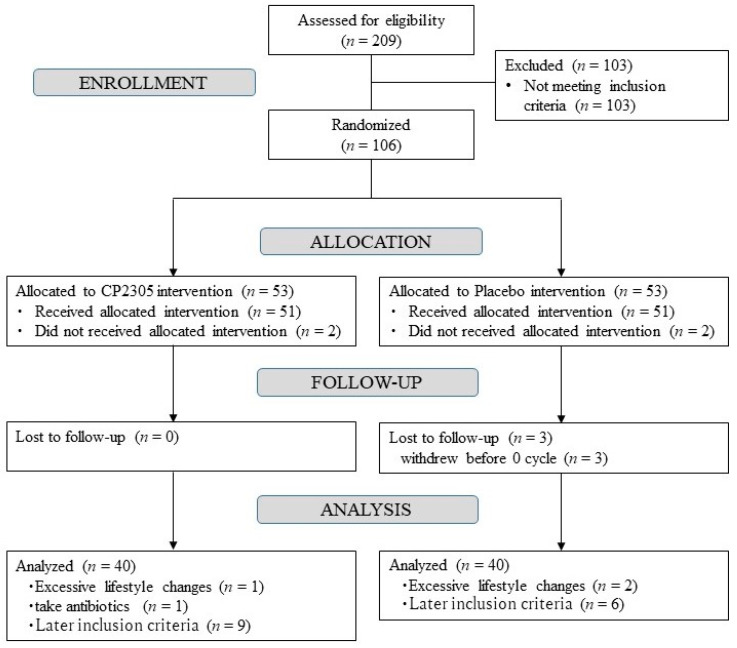
Flow chart of the selection process based on the inclusion/exclusion criteria of the subjects enrolled in the study. The subsequent inclusion criteria are that the diary entry did not meet the inclusion criteria before ingestion. CP2305, *Lactobacillus gasseri* CP2305.

The selection process of the subjects enrolled to this study is shown in Figure 1. Two hundred and nine patients were classified according to the inclusion/exclusion criteria. A total of 103 were excluded, while 106 were considered healthy based on the results of the blood analysis, blood pressure measurement, and self-reported information. The participants were randomly assigned to either the CP2305 (*n* = 51) or placebo (*n* = 51) groups. In the placebo group, one participant withdrew from the study because of personal problems, and two participants withdrew because their menstruation stopped before the first visit.

After the intervention, 19 participants were excluded—15 had an abnormal menstrual cycle before registration depending on their diary, 3 had excessive changes in exercise or dietary habits, and 1 consumed antibiotics. Finally, the analysis was conducted with 80 participants—40 each in the CP2305 and placebo groups.

There were no significant differences in age, BMI; menstrual cycle; menstrual period; levels of E_2_, FSH, and Equol; or SMI total score between the CP2305 and placebo groups before the start of ingestion (Table 2). No adverse events were observed in any of the subjects, including the excluded ones, throughout the trial. The mean compliance rates were 99.7% for the placebo and 99.8% for the CP2305 groups (no significant difference by the χ^2^ test). Medication was ascertained by logbooks, and except for one person who was a regular antibiotic user who was excluded from the analysis, no daily medication was identified.

### 3.2. Effects of CP2305 on Menopausal Symptoms 

**Figure 2:**
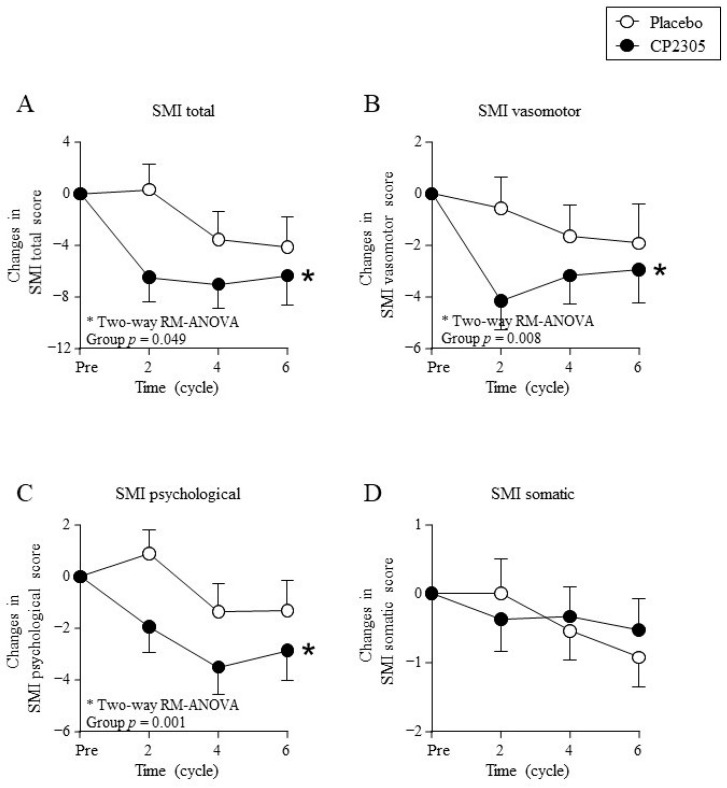
Changes in the Simplified Menopausal Index (SMI) during treatment. Points represent (**A**) changes in SMI total score, (**B**) changes in SMI vasomotor score, (**C**) changes in SMI psychological score, and (**D**) changes in SMI somatic score. * represents statistical difference between groups by two-way repeated measures (RM) ANOVA (detailed statistical results in Supplementary Materials Table S1). The baseline score values, post-intake score values, and percent reduction calculated using these two scores are summarized in Supplementary Materials Table S2. “Pre” indicates the values measured one-cycle before the start of ingestion.

Figure 2 shows changes in the SMI questionnaire scores. The SMI total score (Figure 2A), subscale SMI vasomotor symptom score (Figure 2B), and SMI psychological symptom score (Figure 2C) decreased significantly in the CP2305 group compared with those in the placebo group (two-way ANOVA with repeated measures). Thus, the group that ingested CP2305 for six menstrual cycles showed significantly improved total general symptom scores on the SMI compared with the placebo group. However, no significant difference in the SMI somatic score was detected between the two groups (Figure 2D).

**Figure 3:**
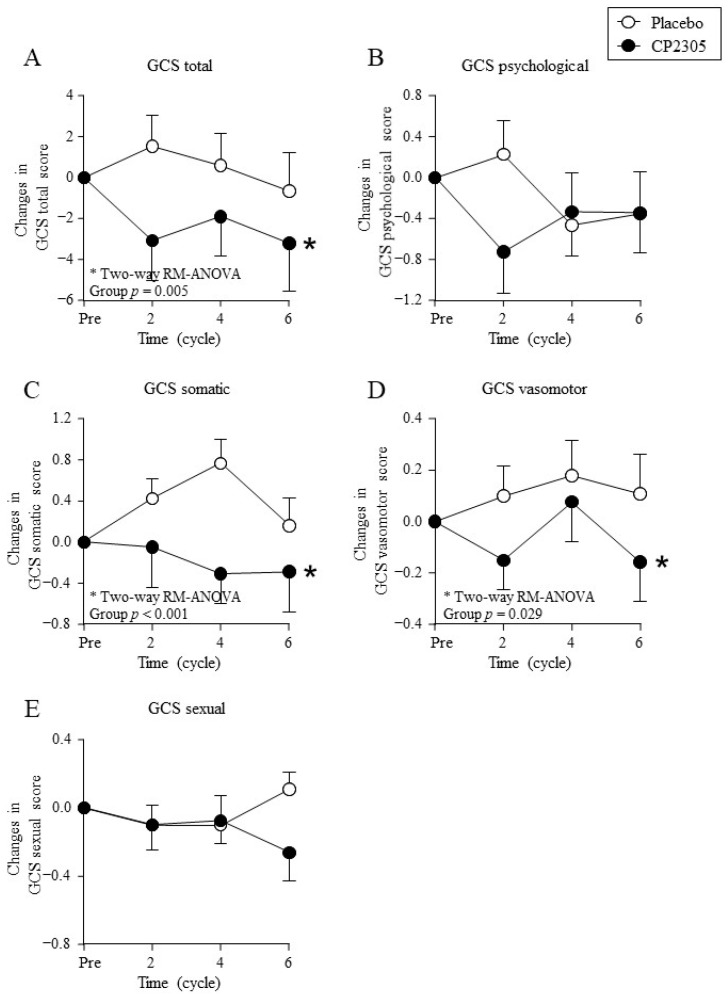
Changes in the Greene climacteric scale (GCS) during treatment. Points represent (**A**) changes in GCS total score, (**B**) changes in GCS psychological score, (**C**) changes in GCS somatic score, (**D**) changes in GCS vasomotor score, and (**E**) changes in GCS sexual score. * represents statistical difference between groups by two-way repeated measures (RM) ANOVA (detailed statistical results in Supplementary Materials Table S1). The baseline score values, post-intake score values, and percent reduction calculated using these two scores are summarized in Supplementary Materials Table S2.

Figure 3 shows the changes in the GCS questionnaire scores. The GCS total score (Figure 3A), subscale GCS somatic symptom score (Figure 3C), and GCS vasomotor symptom score (Figure 3D) decreased significantly in the CP2305 group than those in the placebo group (two-way ANOVA with repeated measures). No significant difference was detected between the two groups in the GCS psychological symptom score (Figure 3B) or GCS sexual score (Figure 3E).

To ascertain the number of women whose symptoms were alleviated, the mean value of the intake period for each individual was subtracted from the baseline value for each individual, which was then tabulated and evaluated. The percentage of women whose symptoms were alleviated by the SMI total score was 75.0% (30 of 40) in the CP2305 group compared to 55.0% (22 of 40) in the placebo group (*p* = 0.059 by χ^2^ test). On the other hand, the percentage of women whose symptoms were alleviated by the GCS total score was 52.5% (21 of 40) in the CP2305 group versus 40.0% (16 of 40) in the placebo group (*p* = 0.262 by χ^2^ test).

### 3.3. Effects of CP2305 Intake on Levels of Reproductive Hormones during the Follicular Phase and Menstrual Cycle

The variation in the concentration of reproductive hormones during the follicular phase and menstrual cycle are shown in Table 3 and Table 4, respectively. Two-way ANOVA with repeated measures revealed that there were no significant differences between the groups in the levels of E_2_, P_4_, FSH, and LH (Table 3). Moreover, the menstrual cycle duration showed no significant differences between the groups (Table 4).

## 4. Discussion

This study assessed the effects of CP2305 on menopausal symptoms in middle-aged women. The analyses suggest that daily administration of CP2305 significantly improves psychological and vasomotor menopausal symptoms, with no effect on the follicular phase levels of reproductive hormones.

The present study showed that the intake of CP2305 may alleviate the most common psychological symptoms, such as irritability, depression, insomnia, and dizziness, as well as vasomotor symptoms, such as hot flashes, chills, excessive sweating, and palpitations, in the SMI scores. It has been reported that the characteristic symptoms experienced by women in Japan are psychological symptoms, such as stiff shoulders, reduced memory, irritability, and depression [32,33]. Therefore, CP2305 may suppress the symptoms that are characteristic of menopausal women in Japan.

To assess symptoms, repeated measures ANOVA was used to evaluate the effect of treatment, as well as the effect of time and the effect of interaction (Supplementary Materials Table S1). For the SMI total score, the effect of time was significant (*p* < 0.001), and for the GCS total score, it was not significant, but slightly observed (*p* = 0.147). The reduction in scores over time suggests that there is a placebo effect in both groups. However, as the treatment effect was detected even after considering and separating the time effect and the interaction effect, CP2305 ingestion is considered to significantly alleviate various symptoms compared to placebo ingestion.

A study on premenopausal symptoms suggested that the intake of CP2305 may alleviate the autonomic and psychological symptoms that are unique to women [23]. The efficacy of *Lactobacillus acidophilus* YT1 in menopausal women has been shown to alleviate symptoms and improve quality of life [34]. There are still few reports of microbial materials that have shown efficacy in menopausal symptoms, and the fact that CP2305 was evaluated for symptoms with a validated questionnaire suggests that it could be an option as a daily food for women suffering from menopausal symptoms, although the effect may not be great and the explanation may be inadequate.

A depressed mood during the transition to menopause was reported to be associated with increased levels of FSH and LH, and increased variability of E_2_, FSH, and LH [35]. Changes in E_2_ levels can lead to changes in the serotonergic and noradrenalinergic systems, and dysregulation of the monoaminergic pathways of the central nervous system may lead to a depressive mood [36]. In contrast, no significant changes in the levels of female hormones in the blood during the follicular phase were detected. A study of CP2305 and premenstrual symptoms showed that CP2305 had no effect on the follicular phase, but that it had an effect on fluctuations in female hormones during the luteal phase [23]. These results suggest that CP2305 may have the greatest effects on repairs during the phases that show fluctuations in female hormones (from the ovulatory to luteal phases), but may not affect the basic steady state. It has been suggested that large fluctuations in E_2_ levels may be involved in psychological symptoms during menopause [37]. As E_2_ fluctuates significantly in the luteal phase rather than in the follicular phase, CP2305 may relieve symptoms by suppressing the fluctuation in E_2_ levels in the luteal phase rather than in the basal stationary phase.

Thus, CP2305 may suppress mental symptoms via endogenous pathways. Another route is circulatory exogenous E_2_ that is predominantly produced by the intestinal microflora [14]. The gut microbiota can regulate E_2_ via the secretion of β-glucuronidase, an enzyme that uncouples E_2_ to its active form. This study failed to evaluate changes in the gut microflora due to CP2305 consumption; however, it suggested that CP2305 intake increased the diversity of the gut microflora [38]. Therefore, CP2305 may regulate the intestinal microbiota to increase extrinsic E_2_ or tryptophan metabolites produced by the microflora.

There are several theories regarding the mechanism, but vascular motor symptoms are a typical sign of imbalance in the autonomic nerve activity. As the oral administration of CP2305 has been suggested to affect the autonomic nerve activity [24,39], CP2305 may act on the body temperature center and suppress the appearance of vasomotor symptoms. In contrast, calcitonin gene-related peptide microbiota (CGRP) is known to have a vasodilatory effect as a hormone secreted into the blood. Neuropeptides such as CGRP are expressed at all levels of the microflora–intestine–brain axis, and have been suggested to play an important role in bidirectional signaling between the intestine and brain [40]. Therefore, it is possible that CP2305 functions along this axis and promotes its action.

Dietary habits are important for maintaining quality of life in middle-age women. One typical dietary component that alleviates menopausal symptoms is phytochemicals such as soy isoflavones [41,42,43]. In particular, genistein contained in isoflavones has a female hormone-like effect, and its continuous intake in combination with vitamin D and calcium for one or two years has been reported to improve bone metabolism, hot flashes frequency, and glucose metabolism [41,42,43]. Considering that dietary patterns were not investigated and that the intake was approximately 0.5 years in this study, it is thought that dietary isoflavone aglycones have little effect on menopausal symptoms, especially with regard to hot flashes. On the other hand, dietary isoflavone glycosides are unknown and will be the subject of a future study.

Equol, which is produced by the conversion of isoflavones by the intestinal bacteria, also has estrogen-like effects. It is known that daidzin, a type of isoflavone, is converted to daidzein by β-glucosidase of *Bifidobacterium*, *E. coli*, and lactic acid bacteria [44]. Furthermore, it has been reported that daidzein is converted to equol by the family *Coriobacteriaceae* and the genera *Lactobacillus*, *Lactococcus*, and *Bifidobacterium* [45]. On the other hand, CP2305 treatment has been reported to result in an increase in *Bifidobacterium* and an increase in intestinal bacterial diversity [35]. Therefore, understanding dietary isoflavones and the variation in the intestinal microbiota is interesting and should be one of the future plans for elucidating the mechanism of action of CP2305.

Phytoestrogens bioactivated by intestinal bacteria act as exogenous estrogens and assist in the age-related decline of endogenous estrogens. In addition, the HPA and HPG axes, which are homeostatic mechanisms in the host, interact with each other, and an excessive stress response reduces endogenous estrogen production. Therefore, diet and exercise regimens that maintain an appropriate stress response are important. In addition, evening primrose oil, which is high in omega-3 essential fatty acids, has been reported to improve psychological symptoms during menopause [46]. Omega-3s can have an anti-inflammatory effect by directly acting on immune cells and indirectly affecting eicosanoid synthesis [47]. Thus, the combination of the Mediterranean-style diet and a plant-based diet featuring phytonutrients and lipids with gut-modifying compounds, such as prebiotics and paraprobiotics, is an attractive strategy for comfort during the female menopause. The comprehensive combination of these diets may help reduce fluctuations in hormonal balance and menopause-specific symptoms, and may contribute to the promotion of women’s empowerment.

The main limitation of this study is that the precise effects of hormones could not be detected due to possible errors in quantification and large fluctuations in the levels of hormones between individuals. Additionally, a longer ingestion period of CP2305 could have revealed more differences between the groups. This is an issue for future research.

Furthermore, to elucidate the mechanism of action of CP2305 on hormonal fluctuations, the composition of the enteric flora and E_2_ metabolism will be studied. Moreover, stress hormones, such as cortisol, and autonomic activity will be evaluated to determine the basis of the vasomotor effects of CP2305.

## 5. Conclusions

This study shows that consumption of *Lactobacillus gasseri* CP2305 improves mild psychological symptoms that are unique to menopausal women, and improves the most common vasomotor symptoms, such as hot flashes. These findings provide new insights into the role of CP2305 in modulating symptoms in women.

Further studies will be conducted to evaluate the effectiveness of *Lactobacillus gasseri* CP2305 on the psychological health of women at various stages, and to elucidate the psychological mechanism. Based on the data obtained from such analyses, the consumption of CP2305 may be used as an alternative treatment to alleviate menopausal symptoms.

## Figures and Tables

**Table 1:** Experimental schedule.

Variables	Time (Menstrual Cycles)								
	Pre-2	Pre-1	0	1	2	3	4	5	6
Tablet intake			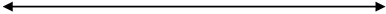						
Questionnaires	●	●			●		●		●
Blood sampling	●	●			●		●		●
Diary		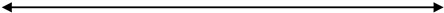							

“Pre” indicates the values measured one cycle before the start of ingestion. Arrows represent once-a-day continuous intake or diary entries, and closed circles represent spot events.

**Table 2:** Participant demographics.

Parameter	Placebo (*n* = 40)	CP2305 (*n* = 40)	*p*-Value
Age (years)	44.6 ± 0.5	45.5 ± 0.5	0.19
BMI (kg/m^2^)	22.5 ± 0.4	22.6 ± 0.4	0.90
Body fat percentage	30.4 ± 1.0	30.3 ± 1.1	0.93
Daily alcohol consumption	2/38	3/37	0.64
Smoking	0/4/4/32	0/2/3/35	0.62
Menstrual cycle (day)	28.2 ± 0.6	28.6 ± 0.5	0.64
Menstrual period (day)	6.0 ± 0.2	5.7 ± 0.2	0.34
E_2_ (pg/mL)	65.4 ± 11.4	59.1 ± 9.6	0.67
FSH (mIU/mL)	13.8 ± 1.7	13.6 ± 1.1	0.94
Equol production	10/30	15/25	0.23
SMI total score	46.2 ± 1.7	46.7 ± 1.9	0.83
POMS fatigue score	13.8 ± 0.7	12.0 ± 0.6	0.052
POMS vigor score	8.3 ± 0.6	6.9 ± 0.6	0.11

Data are presented as mean ± SEM and are analyzed using Student’s *t*-tests. The χ^2^ test is used for the analysis of daily alcohol consumption, smoking, and Equol production. CP2305: *Lactobacillus gasseri* CP2305; BMI: body mass index; E_2_: estrogen; FSH: follicle-stimulating hormone; SMI: Simplified Menopausal Index; POMS: Profile of Mood States; SEM: standard error of the mean.

**Table 3:** Concentration of reproductive hormones during the follicular phase.

Parameters	CP2305 Group	Placebo Group	*p*-Value *
E_2_ (pg/mL)			
Baseline	59.13 ± 9.55	65.40 ± 11.36	
Cycle 2	65.75 ± 11.22	62.03 ± 10.83	
Cycle 4	76.67 ± 12.76	71.03 ± 13.64	
Cycle 6	65.24 ± 11.36	66.54 ± 10.03	
Cycle 2-Baseline	6.63 ± 12.90	−3.38 ± 16.39	
Cycle 4-Baseline	20.41 ± 13.49	4.54 ± 12.96	
Cycle 6-Baseline	9.05 ± 11.77	6.89 ± 12.96	0.36
P_4_ (ng/mL)			
Baseline	0.21 ± 0.05	0.43 ± 0.28	
Cycle 2	0.33 ± 0.11	0.18 ± 0.05	
Cycle 4	0.63 ± 0.34	0.56 ± 0.37	
Cycle 6	0.13 ± 0.02	0.10 ± 0.01	
Cycle 2-Baseline	0.11 ± 0.10	−0.25 ± 0.24	
Cycle 4-Baseline	0.42 ± 0.34	0.12 ± 0.48	
Cycle 6-Baseline	−0.09 ± 0.05	−0.05 ± 0.02	0.13
FSH			
Baseline	13.63 ± 1.06	13.77 ± 1.65	
Cycle 2	14.51 ±1.47	12.77 ± 1.30	
Cycle 4	13.51 ± 1.23	16.35 ± 2.08	
Cycle 6	16.81 ± 2.19	13.06 ± 1.77	
Cycle 2-Baseline	0.88 ± 1.72	−1.00 ± 1.91	
Cycle 4-Baseline	−0.32 ± 1.30	2.99 ± 1.48	
Cycle 6-Baseline	3.06 ± 2.00	0.13 ± 1.93	0.75
LH			
Baseline	5.46 ± 0.42	5.13 ± 0.59	
Cycle 2	7.50 ± 1.25	5.01 ± 0.45	
Cycle 4	6.55 ± 1.14	7.95 ± 1.20	
Cycle 6	7.84 ± 1.15	5.54 ± 0.66	
Cycle 2-Baseline	2.04 ± 1.13	−0.12 ± 0.61	
Cycle 4-Baseline	1.01 ± 1.09	2.93 ± 0.95	
Cycle 6-Baseline	2.25 ± 0.96	0.70 ± 0.73	0.45

“Baseline” indicates the values measured one-cycle before the start of ingestion. Data are presented as mean ± SEM. * The changes between the placebo and CP2305 groups are analyzed with repeated measures of two-way ANOVA. E_2_: estradiol; P_4_: progesterone; FSH: follicle-stimulating hormone; LH: luteinizing hormone.

**Table 4:** Transitions in menstrual cycle days.

Parameters	CP2305 Group	Placebo Group	*p*-Value *
Baseline	28.6 ± 0.5	28.2 ± 0.6	
Cycle 1	28.2 ± 1.2	28.3 ± 0.9	
Cycle 2	29.1 ± 1.0	27.2 ± 0.7	
Cycle 3	29.5 ± 1.3	28.9 ± 1.2	
Cycle 4	27.2 ± 0.6	30.6 ± 1.9	
Cycle 5	29.2 ± 1.1	27.9 ± 1.7	
Cycle 6	29.2 ± 0.9	28.0 ± 0.7	0.29

“Baseline” indicates the values measured one-cycle before the start of ingestion. Data are presented as mean ± SEM. * The changes between the placebo and CP2305 groups are analyzed with repeated measures of two-way ANOVA.

## Data Availability

The data presented in this study are available from the corresponding author upon reasonable request.

## Supplementary Materials

The following supporting information can be downloaded at: https://www.mdpi.com/article/10.3390/nu14091695/s1, Table S1. Results of statistical analysis of questionnaires and reproductive hormones; Table S2. Results for questionnaire scores and percent decrease.
