# Supplementary material for: Effects of Lactobacillus gasseri CP2305 on Mild Menopausal Symptoms in Middle-Aged Women
Source: Nutrients. 2022 Apr 19;14(9):1695. doi: 10.3390/nu14091695 (PMC9101532; doi:10.3390/nu14091695)
Supplement: Supplementary file 1 [file nutrients-14-01695-s001.zip › nutrients-1659855-supplementary.pdf]

Table S1. Results of statistical analysis of questionnaires and reproductive hormones.

Parameters			Two-way repeated-measures ANOVA analysis *p*-value		
			Treatment	Time	Interaction (Treatment × Time)
Figure 2	A	SMI total	0.049 *	<0.001 *	0.740
	B	SMI vasomotor	0.008 *	0.003 *	0.540
	C	SMI psychological	0.001 *	0.001 *	0.236
	D	SMI somatic	0.920	0.020 *	0.525
Figure 3	A	GCS total	0.005 *	0.147	0.320
	B	GCS psychological	0.219	0.115	0.643
	C	GCS somatic	<0.001 *	0.867	0.053
	D	GCS vasomotor	0.029 *	0.806	0.283
	E	GCS sexual	0.239	0.259	0.112
Table 3		E_2	0.362	0.295	0.818
		P_4	0.126	0.940	0.555
		FSH	0.754	0.150	0.772
		LH	0.445	0.007 *	0.987

* significant difference ($p < 0.05$). Abbreviations: GCS: Greene climacteric scale; SMI: Simplified Menopausal Index; E_2: estradiol; P_4: progesterone; FSH: follicle-stimulating hormone; LH: luteinizing hormone.

Table S2. Results for questionnaire scores and percent decrease.

Parameters	Treatment	Baseline	Cycle 6	Rate of decrease
SMI total	Placebo	46.2 ± 1.7	42.3 ± 2.2	91.6%
	CP2305	46.7 ± 1.9	40.7 ± 2.3	87.1%
SMI vasomotor	Placebo	18.7 ± 1.2	16.9 ± 1.2	90.2%
	CP2305	18.7 ± 0.9	15.9 ± 1.0	85.0%
SMI psychological	Placebo	18.2 ± 1.1	17.1 ± 1.1	93.9%
	CP2305	19.7 ± 1.2	16.9 ± 1.3	86.1%
SMI somatic	Placebo	9.2 ± 0.5	8.3 ± 0.6	90.1%
	CP2305	8.4 ± 0.5	7.8 ± 0.6	93.9%
GCS total	Placebo	10.1 ± 0.7	9.6 ± 1.6	95.0%
	CP2305	11.9 ± 0.8	8.7 ± 2.1	73.4%
GCS psychological	Placebo	5.1 ± 0.4	4.9 ± 0.4	96.5%
	CP2305	5.3 ± 0.4	5.0 ± 0.4	95.3%
GCS somatic	Placebo	3.0 ± 0.3	3.1 ± 0.3	105.4%
	CP2305	4.1 ± 0.4	3.8 ± 0.5	92.3%
GCS vasomotor	Placebo	0.9 ± 0.1	0.9 ± 0.1	108.1%
	CP2305	1.1 ± 0.1	0.9 ± 0.1	85.2%
GCS sexual	Placebo	1.1 ± 0.1	1.2 ± 0.1	110.5%
	CP2305	1.5 ± 0.1	1.2 ± 0.1	80.7%

Abbreviations: GCS: Greene climacteric scale; SMI: Simplified Menopausal Index; CP2305, *Lactobacillus gasseri* CP2305.
